# Evaluation of semen DNA integrity and related parameters with COVID-19 infection: a prospective cohort study

**DOI:** 10.1186/s12985-023-02192-y

**Published:** 2023-09-28

**Authors:** Shuibo Shi, Hongji Hu, Jiayao Wang, Xueming Huang, Jianhao Li, Dongshui Li

**Affiliations:** 1 Department of Assisted Reproduction, the First Affiliated Hospital of Nanchang University, Yongwai Street 17, Nanchang City, Jiangxi Province China; 2 Department of Clinical Laboratory, the First Affiliated Hospital of Nanchang University, Yongwai Street 17, Nanchang City, Jiangxi Province China

**Keywords:** COVID-19, Fertility, Sperm parameters, DFI

## Abstract

**Background:**

In the context of Corona Virus Disease 2019 (COVID-19) global pandemic**,** Its impact on male reproductive function should be concerned.

**Methods:**

Our study is a prospective cohort study that recruited participants infected or uninfected with COVID-19 between December 2022 and March 2023. All laboratory tests and questionnaire data were completed at the First Affiliated Hospital of Nanchang University. A total of 132 participants were enrolled, with 78 COVID-19 positive patients as the positive group and 54 COVID-19 negative participants as the negative group. Semen quality was assessed by the fifth World Health Organization criteria. The general characteristics of semen samples were assessed using CASA (computer-assisted sperm analysis). DNA damage and the high density stainability was assessed by sperm chromatin structure analysis (SCSA) based on flowcytometry.

**Results:**

The sperm concentration, progressive motility and motility in COVID-19 negative group were significantly higher than positive group. In the following DNA damage analysis, a remarkably lower sperm DNA fragmentation index (DFI) in the COVID-19 negative group. In the positive group, unhealthy lifestyles had no significant effect on semen parameters, DNA fragmentation and nuclear compaction.

**Conclusions:**

After excluding the interference of unhealthy lifestyle, the COVID-19 infection can have a significant impact on the quality of semen, especially the DFI,. Therefore, it shows that COVID-19 can adversely affects male fertility, and this result provides advisory guidance for clinicians.

## Introduction

Severe Acute Respiratory Syndrome Coronavirus 2 (SARS-COV-2) is a single-stranded RNA virus belongs to the coronavirus family. Prior to the SARS-COV-2 outbreak epidemic, coronaviruses caused higher pathogenic and fatal diseases, including Middle East Respiratory Syndrome coronavirus (MERS-CoV) and Severe Acute Respiratory Syndrome coronavirus (SARS-CoV) [1]. Several studies have shown that the virus not only affects the respiratory system but also causes pathological changes in other organs, such as the kidney [2], cardiac [3], liver, brain [4], and testes. The presence of SARS-COV-2 in saliva, respiratory fluids, blood, urine, and feces has been reported, and there is increasing evidence of SARS-COV-2 infection and inflammation in semen or testes [5, 6]. The testes and epididymis of patients who died from COVID-19 exhibited pathological changes such as interstitial edema, congestion, germ cell destruction, thinning of germinal tubules, and increased spermatogenic epithelial detachment [5, 7], which may further reveal the negative impact of COVID-19 on male fertility.

In some studies, it has been suggested that persistent fever during viral infection may disrupt the blood-testis barrier (BTB) [8], while the finding of SARS-COV-2 in the endothelial cells of the BTB offers the possibility of virus invasion [6]. In addition, Angiotensin Converting Enzyme 2 (ACE2), which has a high affinity with SARS-COV-2, is also highly expressed in the male reproductive system, especially in spermatogonia [9], which may reveal the mechanism of entry into the male reproductive organs. Studies have shown that the immune response generated in testicular tissue adversely affects sperm production, which may impair male hormonal function and fertility [10, 11].

In previous studies on the impact of COVID-19 on male reproductive system, positive results were reported. For instance, Holtmann et al. found statistically significant reductions in sperm concentration, total sperm count, total number of progressive sperm, and total number of motile sperm in 20 moderately infected COVID-19 patients [12]. Similarly, Ma et al. conducted a study on 12 COVID-19 patients and found normal sperm parameters and low DFI in eight patients, while low sperm motility and high sperm DFI were observed in four patients [13]. However, it is important to note that the sample size of these previous studies were small, despite the use of exclusion and screening criteria, they may still have lacked control of some confounders and the effect on sperm quality in infected patients needs to be confirmed in more studies with clinical samples. In our study, we aimed to analyze and compare the DFI and other parameters of sperm between the COVID-19 positive and negative groups and to exclude some confounders, after excluded the effects of disease and medicine, we investigated unhealthy lifestyle habits in the positive group.

## Methods

### Study design

This study was conducted by the First Affiliated Hospital of Nanchang University and approved by the Ethics Committee. As a prospective cohort study, in order to calculate the sample size required for our study, in the Gpower software, with two tails, input parameters of effect size = 0.6, alpha level = 0.05, statistical power = 0.8, the minimum required sample size was determined to be 47.Between December 2022 and March 2023, we recruited participants from the community who volunteers to participate in the study, provide semen analysis and had records of COVID-19 test. By stratified random sampling method, a total of 132 male participants were included to observe the effect of COVID-19 on male sperm. Male participants aged 30.58 ± 5.16 years old with a median age of 30 (IQR 27–34) years, and collect unhealthy lifestyle information in positive group. We have included four unhealthy lifestyle habits, smoking, drinking alcohol, staying up late and sedentariness as confounders. In the questionnaire, we defined smoking habits as smoking at least 10 cigarettes daily, alcohol consumption habits as a weekly alcohol intake over 150 g, staying up late as fall asleep after 11:00 pm [14] and sedentariness as sitting without movement for more than four hours due to work reason or habits. All participants had no history of cryptorchidism, chronic disease, infectious disease, varicocele surgery, or testicular surgery. Moreover, all patients did not take drugs affecting androgen levels and their androgen levels were within normal range. Among the 132 participants, 78 patients had COVID-19 within the last three months (COVID-19 positive group), and confirmed by two positive polymerase chain reaction tests (throat swab sampling). The other 54 participants were never infected with COVID-19 (COVID-19 negative group). Among the 78 patients, the symptoms of COVID-19 infection were between mild and moderate, and no patients were sent to the hospital for treatment. After they complete the questionnaire, semen samples were obtained through masturbation at our andrology research center and conducted further analysis. According to the fifth World Health Organization (WHO) criteria [15], three to seven days sexual abstinence duration were required.

### Semen analysis

The Andrology Research Center in the First Affiliated Hospital of Nanchang University have completed the semen quality assessment. All analyses in the laboratory were performed according to the fifth WHO criteria. The semen samples received by the andrology clinic were placed in an incubator at 37℃ for 30 min to liquefaction, and the liquefied samples were treated in a laminar sterilization cabinet. The general characteristics of semen samples were assessed using computer-assisted sperm analysis (CASA), such as total sperm count (× 10^6^ per ejaculate), Concentration(× 10^6^ ml^−1^), Progressive motility (%), non-progressive motility (%), and immotility (%). The Semen assessment was performed according to the fifth WHO laboratory manual for the examination and processing of human semen. sperm motility was assessed as three types: progressive(sperm motile and active), non-progressive(sperm motile but inactive) and immotility(Sperm do not move at all).

In order to check the DNA and nuclear compaction of sperm, the DNA fragmentation index (DFI) and sperm High DNA stainability (HDS) were detected by sperm chromatin structure analysis (SCSA) based on flowcytometry. DFI and HDS ≤ 15% is considered normal, when they are more than 15%, means the DNA and nuclear compaction damaged, as abnormol.

### Statistical analysis

The data analysis of this study was mainly implemented through SPSS (IBM SPSS Statistics 25). To compare differences in DFI and other parameters between the COVID-19 positive and negative groups,the parametric method was used for measurements that conformed to a normal distribution, and the independent sample test (t‐table value) method was used to compare measurements from two independent groups. The nonparametric method or Mann–Whitney U test (Z-table value) method is used for measurements that do not conform to normal distribution. After grouping the ages by median, multivariable logistic regression analysis were used by calculating the odds ratio and its 95% confidence interval to ascertain the risk factors of DFI abnormality as control of confounders. Two tailed p values ≤ 0.05 was considered statistically significant. Additionally, the results of this study were completed through the use of frequency tables and descriptive statistics.

## Results

In our research included 132 participants, and the effect of lifestyles on sperm quality were analyzed in positive group. Compared to the negative group, the mean age of positive group (30.85 ± 4.58,30.81 ± 5.71; respectively) was observed not statistically significant. In the completed analysis of sexual abstinence days, semen volumes, total sperm count and sperm concentration, the sperm concentration in COVID‐19 negative group were significantly higher than positive group. Sperm motility was significantly higher in COVID-19 negative group than positive group, especially progressive motility. Furthermore, the sperm DFI was significantly higher in the positive group, sperm HDS was not remarkably lower in the COVID‐19 negative group (Table 1). In the COVID-19 positive group, the number of men with unhealthy lifestyles in the positive group were shown in Table 2 and no significant differences of semen quality parameters in the men with and without unhealthy lifestyles (Table 3). Multivariable logistic regression analyses showed that COVID-19 positive (OR = 6.760, 95% CI = 3.009–15.187) was a major risk factor for abnormal DFI (Table4).

**Table 1:** Comparison of semen parameters between COVID-19 positive and negative groups

Variable	Positive (n = 78)		Negative (n = 54)		t/z	*P*-value
Age (year)	30.81 ± 5.71		30.85 ± 4.58		0.04	0.966
Sexual abstinence (day)	4.41 ± 1.83		4.59 ± 1.67		− 0.54	0.585
Volume (ml)	3.38 ± 1.73		3.01 ± 1.46		− 1.19	0.232
Total sperm count (× 10^6^ per ejaculate)	88.16 ± 63.51		102.83 ± 73.86		− 1.15	0.252
Concentration (× 10^6^ ml^−1^)	28.62 ± 21.74		37.48 ± 31.01		− 2.03	**0.042**
Progressive motility (%)	32.17 ± 15.09		36.75 ± 12.41		2.26	**0.027**
Non-progressive motility (%)	6.04 ± 3.94		7.72 ± 5.79		−1.83	0.067
Motility (%)	38.58 ± 17.01		44.53 ± 14.87		−2.08	**0.037**
DNA fragmentation index (DFI, %)	21.97 ± 13.35		13.01 ± 7.87		− 4.68	** < 0.001**
High DNA stainability (HDS,%)	18.27 ± 7.40		15.56 ± 6.40		− 1.52	0.129

Bold values indicate statistically significant differences in the parameters compared between the two groups (p < 0.05). The sperm concentration (× 10^6^ ml^−1^), motility (%), and progressive motility (%) of the positive group were significantly lower than those of the negative group. In terms of the DNA fragmentation index (DFI, %), the positive group was remarkably higher than the negative group

**Table 2:** Distribution of the four lifestyles in the COVID-19 positive group

Characteristics	Total positive n = 78 (%)
Smoking history	
Yes	29 (37.2)
No	49 (62.8)
Drinking history	
Yes	8 (10.3)
No	70 (90.7)
Sleeping late history	
Yes	57 (73.1)
No	21 (26.9)
Sedentariness history	
Yes	58 (74.4)
No	20 (25.6)

**Table 3:** Comparison of sperm parameters within each lifestyle

Characteristics	Volume (ml)	Total sperm count(× 10^6^ per ejaculate)	Concentration (× 10^6^ ml^−1^)	Progressive motility (%)	Non-progressive motility (%)	Motility (%)	DNA fragmentation index (DFI, %)	High DNA stainability (HDS,%)
Smoking history								
Yes	3.33 ± 1.80	71.62 ± 52.92	25.78 ± 21.03	32.01 ± 12.13	6.06 ± 5.09	38.93 ± 15.44	21.17 ± 11.37	20.01 ± 8.21
No	3.34 ± 1.66	93.91 ± 66.02	30.17 ± 21.89	31.12 ± 16.50	6.17 ± 4.02	37.38 ± 18.28	22.59 ± 1.68	17.38 ± 6.95
*P*-value	0.766	0.122	0.291	0.703	0.667	0.77	0.699	0.766
Drinking history								
Yes	3.25 ± 2.73	100.92 ± 87.93	35.02 ± 25.08	31.09 ± 17.84	6.87 ± 4.26	40.57 ± 23.26	23.13 ± 10.35	17.33 ± 8.66
No	3.33 ± 1.61	85.13 ± 60.06	28.10 ± 21.29	31.44 ± 14.97	6.06 ± 4.40	37.62 ± 16.79	22.01 ± 13.19	18.36 ± 7.37
*P*-value	0.367	0.794	0.468	0.965	0.542	0.739	0.464	0.845
Sleeping late history								
Yes	3.30 ± 1.67	84.96 ± 62.47	28.31 ± 21.85	30.47 ± 15.63	5.84 ± 4.46	36.71 ± 17.93	22.99 ± 13.40	18.57 ± 7.03
No	3.47 ± 1.83	91.69 ± 62.47	30.05 ± 21.21	34.41 ± 13.36	6.98 ± 4.05	41.64 ± 14.97	19.33 ± 11.07	17.23 ± 8.75
*P*-value	0.62	0.57	0.66	0.236	0.217	0.256	0.139	0.345
Sedentariness history								
Yes	3.29 ± 1.72	73.16 ± 59.74	24.17 ± 19.72	24.17 ± 19.72	29.90 ± 15.94	6.40 ± 6.01	37.30 ± 18.89	19.57 ± 6.24
No	3.24 ± 1.70	89.98 ± 63.56	30.07 ± 22.67	30.07 ± 22.67	30.72 ± 15.66	5.65 ± 3.67	36.47 ± 17.72	18.14 ± 7.36
*P*-value	0.655	0.267	0.261	0.717	0.94	0.913	0.864	0.162

There was no statistically significant effect of the four lifestyles (smoking, drinking, sleeping late, and sedentariness) on sperm parameters (p > 0.05)

**Table 4:** Multivariable logistic regression analysis of risk factors of DFI abnormality patients

Characteristics	Multivariate analysis	
	OR (95%CI)	*P*
Age		
≤ 30	Reference	
> 30	1.799 (0.812–3.986)	0.148
COVID-19 infection		
No	Reference	
Yes	6.760 (3.009–15.187)	**< 0.001**
Smoking history		
No	Reference	
Yes	0.569 (0.235–1.379)	0.212
Drinking history		
No	Reference	
Yes	1.434 (0.365–5.638)	0.606
Sleeping late history		
No	Reference	
Yes	1.780 (0.683–4.641)	0.238
Sedentariness history		
No	Reference	
Yes	0.423 (0.175–1.020)	0.055

## Discussion

Our findings show that the DFI was remarkably higher in the COVID-19 positive group (P < 0.001), which indicates a damaging effect on sperm DNA. Haghpanah et al. [16] stated that sperm DFI may serve as a promising and important factor for male infertility due to COVID-19 infection. Likewise, according to the latest WHO standards [17], sperm DFI can be used as an important complement to assess male fertility. Furthermore, in agreement with the findings of Caliskan et al. [18] observed a negative correlation between sperm DFI, sperm concentration and percentage of motility in the analysis of sperm samples from 743 infertile men. In the study by Dipankar et al. [19], all 30 COVID-19 positive participants included in the survey had a DFI more than 30%. these results further support that COVID-19 affects male infertility and plays an important role of DNA damage in sperm. In addition, we investigated the impact of unhealthy lifestyle on sperm quality in the COVID-19 positive group, but no statistically significant differences were observed. The conclusions of Donders et al. [20] are consistent with ours, they found that smoking and BMI were not associated with any sperm quality parameter in a multiple regression analysis of infected patients. In a study on the effect of lifestyle changes on semen quality. Although there were studies proved that unhealthy lifestyles impact semen quality [21], these results and our findings both indicated that sperm quality did not show significant differences in postive group, whereas the adverse effect of COVID-19 on sperm quality was further validated and a positive COVID-19 is the only risk factor of abnormal DFI.

Multiple studies have shown that SARS-COV-2 can infect the testes. Stanley et al. [22] pointed out that besides ACE2 expressed in the testis, another molecule, transmembrane serine protease 2 (TMPRSS2), is also expressed in testicular tissue and spermatozoa. It induces conformational changes by cleaving the viral S protein, thereby fusing the virus to the host cell membrane. In the study of Koch et al. [23], SARS-CoV-2 can invade target cells through the rapid pathway in TMPRSS2^+^ cells and the slow pathway in TMPRSS2 ^+^ cells. However, ACE2 lacked co-expression with TMPRSS2 in testicular tissues, and the association between them deserves further investigation.

In our study, sperm concentration, and motility were significantly lower in the COVID-19 positive group. It has been reported that COVID-19 induced fever may impair spermatogenesis and lead to decreased sperm quality [24], but this conclusion still faces challenges, In the prospective study of 120 individuals included by Donders et al. [20] shown no significant effect of the severity of infection and fever on sperm was observed. In addition, this analysis revealed a short-term decrease in sperm concentration (P < 0.003) and progressive motility (P < 0.02) in infected patients. According to available clinical data, most infected patients develop varying degrees orchitis and genital tract inflammation, additionally, the overproduction of cytokines that regulate the immune response (IL-6, etc.) induced by a viral infection can lead to leukocyte infiltration in the interstitium of testis, resulting in autoimmune response and formation of anti-sperm antibodies (ASA) [25] and autoimmune responses appear to play an important role in the negative effects of COVID-19 on fertility. Ertaş et al. [26] concluded that COVID-19 can significantly reduce sperm concentration and total motility. Analogous results were observed in our study, with statistically significant differences in changes with sperm concentration (P = 0.042), motility (P = 0.037), and progressive motility (P = 0.027) in infected patients compared to negatives. Studies have shown that sperm quality may revert over time in COVID-19 positive patients, but it may takes more than three months. As shown in a prospective longitudinal cohort study by Dipankar et al. [19] comparing the second sampling after 74 days with the first sampling, the number of patients with sperm concentration < 150,000/mL was reduced from fourteen to five, and 30 COVID-19 positive patients with sperm concentration (P < 0.001), viability (P = 0.014), total motility (P = 0.002) and DFI (P < 0.001) were significantly improved, but the quality remained poor. In another prospective study, Enikeev et al. [27] analyzed semen samples of 44 COVID-19 positive patients during hospitalization and three months after discharge and compared them with 44 normal controls. It was observed that positive patients returned to normal levels of all parameters three months after discharge, even in moderate or severe COVID-19 patients. These findings demonstrate that COVID-19 may cause a temporary decrease in sperm quality, gradually recovery or even rehabilitation over time, but it also needs more clinical data to confirm.

Inflammatory responses and oxidative stress (OS) have been proposed as possible mechanisms for the negative effects of COVID-19 on male fertility [28, 29]. OS and inflammation are usually correlated. SARS-CoV-2 can induce inflammatory responses and overproduction of reactive oxygen species (ROS) through immune responses and ultimately lead to OS [30]. Direct evidence is provided by the study of Hajizadeh et al. [31]. In their prospective longitudinal cohort study, the levels of inflammatory markers (IL-1β, IL-6, IL-8, IL-10, TGF-β, INF-α, and INF-γ) in the semen of the COVID-19 positive group were significantly higher than those of the control group from the first sampling to up to 60 days of follow-up thereafter (p < 0.05). In a study on the treatment of varicocele-induced decline in semen quality with medication, Melissa officinalis was found to effectively improve sperm count, motility, and chromatin structure [32], indicating the protective effect of antioxidant on the male reproductive system [33]. Similarly, in the treatment of a unilateral testicular ischemia reperfusion injury model, citral demonstrated a powerful protective effect [34], displaying strong anti-inflammatory and antioxidant effects. This also suggests that anti-inflammatory and antioxidant therapies may have a positive role in the treatment of COVID-19 patients.

The strength of our study was that we compared the semen from negative and positive participants, identified the differences and compared whether unhealthy lifestyles had an effect on positive patients. However, there are some unavoidable limitations in our study. Firstly, due to the specificity of specimens, we lack of pre-COVID-19 sperm to perform pre and post-infection comparisons. Secondly, our study was developed by clinical samples, potential selection bias and measurement error could have adversely affected the conclusions, In addition, due to the limited information on the variables, it was not yet sufficient to conduct sensitivity analysis. Thirdly, more long-term effects of the COVID-19 on male need more observations of the spermatogenic cycle. Therefore, to learn more about the effects of COVID-19 in men, need a long time patients follow to study the underlying mechanisms and find ways to mitigate the impact during and after COVID-19 infections.

## Conclusion

In our study, we found that semen quality can be significantly affected during COVID-19 infection, semen concentration, progressive motility, motility, especially sperm DFI were significantly decrease, which has a greater impact on male fertility, therefore, reproductive advice can be offered to men after a COVID-19 infection to prevent adverse fertility results, for instance, it is not recommended that patients with COVID-19 infections have a pregnancy plan within three months as a spermatogenic cycle.

## Data Availability

The datasets used and analyzed are available from the corresponding author on reasonable request.

## Abbreviations

COVID-19: Corona virus disease 2019
CASA: Computer-assisted sperm analysis
DFI: DNA fragmentation index
HDS: High DNA stainability
MERS-CoV: Middle east respiratory syndrome coronavirus
SARS-CoV: Severe acute respiratory syndrome coronavirus
TMPRSS2: Transmembrane serine protease 2
ACE2: Angiotensin converting enzyme 2
BTB: Blood-testis barrier
BMI: Body mass index
ASA: Anti-sperm antibodies
OS: Oxidative stress
ROS: Reactive oxygen species
DNA: Deoxyribo nucleic acid
